# Oceanographic features and limited dispersal shape the population genetic structure of the vase sponge *Ircinia campana* in the Greater Caribbean

**DOI:** 10.1038/s41437-020-0344-6

**Published:** 2020-07-22

**Authors:** Sarah M. Griffiths, Mark J. Butler, Donald C. Behringer, Thierry Pérez, Richard F. Preziosi

**Affiliations:** 1 Ecology and Environment Research Centre, Manchester Metropolitan University, Manchester, UK; 2 Department of Biological Sciences, Institute of Environment, Florida International University, North Miami, FL USA; 3 Fisheries and Aquatic Sciences, University of Florida, Gainesville, FL USA; 4 Emerging Pathogens Institute, University of Florida, Gainesville, FL USA; 5 Institut Méditerranéen de Biodiversité et d’Ecologie Marine et Continentale, Aix Marseille Université, Marseille, France

**Keywords:** Genetic variation, Ecology

## Abstract

Understanding population genetic structure can help us to infer dispersal patterns, predict population resilience and design effective management strategies. For sessile species with limited dispersal, this is especially pertinent because genetic diversity and connectivity are key aspects of their resilience to environmental stressors. Here, we describe the population structure of *Ircinia campana*, a common Caribbean sponge subject to mass mortalities and disease. Microsatellites were used to genotype 440 individuals from 19 sites throughout the Greater Caribbean. We found strong genetic structure across the region, and significant isolation by distance across the Lesser Antilles, highlighting the influence of limited larval dispersal. We also observed spatial genetic structure patterns congruent with oceanography. This includes evidence of connectivity between sponges in the Florida Keys and the southeast coast of the United States (>700 km away) where the oceanographic environment is dominated by the strong Florida Current. Conversely, the population in southern Belize was strongly differentiated from all other sites, consistent with the presence of dispersal-limiting oceanographic features, including the Gulf of Honduras gyre. At smaller spatial scales (<100 km), sites showed heterogeneous patterns of low-level but significant genetic differentiation (chaotic genetic patchiness), indicative of temporal variability in recruitment or local selective pressures. Genetic diversity was similar across sites, but there was evidence of a genetic bottleneck at one site in Florida where past mass mortalities have occurred. These findings underscore the relationship between regional oceanography and weak larval dispersal in explaining population genetic patterns, and could inform conservation management of the species.

## Introduction

The spatial distribution of genetic diversity is influenced by both ecological and evolutionary processes, and can be used to infer a number of important characteristics of species, including dispersal, recruitment and gene flow among populations (connectivity). Understanding these processes can help in ecosystem management and conservation (Baums 2008; Almany et al. 2009). In addition, identifying changes in genetic diversity, population bottlenecks and inbreeding is vital in the face of declining populations and environmental change, given their effects on population resilience, adaptive potential and fitness.

In Greater Caribbean marine ecosystems, sponges are abundant, diverse and serve important ecological functions, including cycling of organic material and habitat provisioning (Diaz and Rutzler 2001; Bell et al. 2014; Valentine and Butler 2019). Sponges form a major component of the benthos in many coral reef ecosystems, and can dominate in shallow hard-bottom lagoons (Bertelsen et al. 2009). Despite their prominence and importance, relatively little is known about population structure in Caribbean sponges. Furthermore, in some localities, sponges have suffered mass mortality events (Butler et al. 1995; Wulff 2006), including in the Florida Keys, where recurring die-offs of the sponge community have had numerous negative consequences for associated communities (Butler et al. 1995, 2016; Herrnkind et al. 1997). Increasing our knowledge of the factors driving population structure in sponges is important in understanding their recruitment and dispersal patterns, and may be important for creating ecosystem-based management plans for the region.

In marine systems, population genetic structure is shaped by a dynamic interplay between life history, oceanographic features and demographic stochasticity (Knutsen et al. 2003; Hoffman et al. 2011). As sponges are sessile for the majority of their life cycle, dispersal at the larval stage is crucial in shaping their population structure. The larval phase is generally very short in sponges, ranging from a few hours to a few days (Maldonado and Riesgo 2008), suggesting that philopatry should be high (with a few known exceptions in the hoplitomella larvae; Vacelet 1999). This life history mode can result in more fragmented and isolated populations, as it limits dispersal and thus connectivity among populations (Shanks 2009). However, ocean currents can potentially increase connectivity among distant locations (Chaves-Fonnegra et al. 2015; Richards et al. 2016). Furthermore, strong wave action, predation and discards from commercial sponge fishing produce fragments, which may disperse before reattaching to the substrate (Wulff 1991; Maldonado and Uriz 1999; Butler et al. 2017). However, temporal variation in ocean circulation, as well as demographic stochasticity, can yield unpredictable and chaotic patterns of population structure (Siegel et al. 2008; Castorani et al. 2017; Drury et al. 2018).

To date, empirical population genetics studies have been conducted on only four Caribbean sponge species (*Xestospongia muta*: López-Legentil and Pawlik 2009; de Bakker et al. 2016; Richards et al. 2016; *Callyspongia vaginalis*: DeBiasse et al. 2010; *Cliona delitrix*: Chaves-Fonnegra et al. 2015; *Spheciospongia vesparium*: Griffiths et al. 2020). In general, these studies have found strongly differentiated populations throughout the region, including among sites only tens of kilometres apart in some instances. However, patterns of regional genetic structure often reflect major ocean currents and hydrology (López-Legentil and Pawlik 2009; Chaves-Fonnegra et al. 2015; Richards et al. 2016; Griffiths et al. 2020), and evidence of long-distance dispersal has been found, suggesting irregular transport of larvae, gametes or sponge fragments in ocean currents over large oceanographic distances (DeBiasse et al. 2010; Chaves-Fonnegra et al. 2015). These studies reveal the complexities of sponge population genetics in the Caribbean, highlighting the need for further research on this understudied taxon, including locations that have not yet been sampled.

In this study, we explore population genetic structure in the Caribbean vase sponge *Ircinia campana* (Lamarck 1814), a common species in coral reef and hard-bottom habitats. The reproduction and larval biology of *I. campana* has not yet been described; however, other *Ircinia* species are viviparous, with no true asexual reproduction (through budding or gemmules), and the vase-shaped morphology of *I. campana* is not susceptible to fragmentation during storms. This species is prone to disease outbreaks, and has suffered a number of mass mortality events, the most extensive having occurred in the Florida Keys (Butler et al. 1995). Due to both its prevalence in the region’s benthic communities and its apparent vulnerability, it is important to understand the genetic diversity and structure of *I. campana* in the Greater Caribbean. Here, we aimed to describe the population structure of *I. campana* and explore the effects of mass mortalities on genetic diversity.

## Materials and methods

### Sampling

**Fig. 1:**
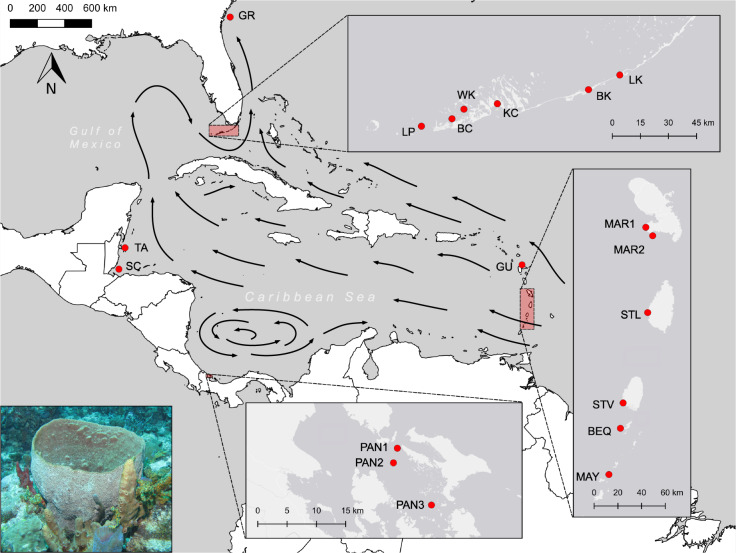
Map showing *Ircinia campana* sampling locations. GR Gray’s Reef, LK Long Key, BK Bamboo Key, KC Kemp Channel, WK Waltz Key, BC Boca Chica Channel, LP Lakes Passage, TA Turneffe Atoll, SC Sapodilla Cayes, PAN1 Panama 1, PAN2 Panama 2, PAN3 Panama 3, MAY Mayreau, BEQ Bequia, STV St. Vincent, STL St. Lucia, MAR1 Martinique 1, MAR2 Martinique 2, GU Guadeloupe. Inset (bottom left): *Ircinia campana* photographed in Bequia (T. Pérez). Arrows show major ocean current patterns in the Caribbean Sea. Basemaps: ESRI, Natural Earth.

We sampled 10–41 *I. campana* individuals at 19 sites throughout the Greater Caribbean (Table 1and Fig. 1) from depths of 1–25 m, resulting in a total sample size of 440 individual sponges. Sponge communities, including *I. campana*, at two of the sampling sites (Bamboo Key and Long Key in the Florida Keys) have previously been affected by mass mortalities associated with cyanobacterial blooms. We cut small fragments of tissue (~1.5 cm^3^) and preserved them in 95% ethanol soon after surfacing. Ethanol was replaced after 24 h to avoid dilution with the seawater held in the sponge tissue.

**Table 1:** *Ircinia campana* sampling locations.

Location	Site name	Site code	*n*	Latitude, longitude	Date (M/YY)
Gray’s Reef National Marine Sanctuary, Georgia, USA	Gray’s Reef	GR	10	31.40480, −80.86677	6/13
Long Key, Florida Keys, USA	Long Key	LK	20	24.81437, −80.83073	7/14
Bamboo Key, Florida Keys, USA	Bamboo Key	BK	16	24.74429, −80.99504	7/14
Kemp Channel, Florida Keys, USA	Kemp Channel	KC	20	24.67687, −81.47577	7/14
Waltz Key, Florida Keys, USA	Waltz Key	WK	17	24.65108, −81.65213	7/14
Boca Chica Channel, Florida Keys, USA	Boca Chica Channel	BC	18	24.60495, −81.71508	7/14
Lakes Passage, Florida Keys, USA	Lakes Passage	LP	20	24.56948, −81.87572	7/14
Turneffe Atoll, Belize	Turneffe Atoll	TA	35	17.54436, −87.82664	4/13
Tom Owen’s Caye, Sapodilla Cayes, Belize	Sapodilla Cayes	SC	39	16.18898, −88.23277	4/13
Bocas Del Toro (1), Panama	Panama 1	PAN1	15	9.32833, −82.22668	7/16
Bocas Del Toro (2), Panama	Panama 2	PAN2	16	9.30604, −82.23275	7/16
Bocas Del Toro (3), Panama	Panama 3	PAN3	11	9.2413, −82.1737	7/16
Mayreau, St. Vincent and The Grenadines	Mayreau	MAY	20	12.64218, −61.37975	5/15
Bequia, St. Vincent and The Grenadines	Bequia	BEQ	27	12.99128, −61.29043	5/15
St. Vincent, St. Vincent and The Grenadines	St. Vincent	STV	40	13.18303, −61.26945	5/15
Anse Chastenet, St. Lucia	St. Lucia	STL	41	13.86413, −61.07882	5/15
Grande Anse d’Arlet, Martinique	Martinique 1	MAR1	17	14.5059, −61.0932	5/15
Diamond Rock, Martinique	Martinique 2	MAR2	26	14.4426, −61.04013	5/15
Port-Louis, Grande-Terre, Guadeloupe	Guadeloupe	GU	32	16.46233, −61.53062	5/15

*n* number of individuals successfully genotyped.

### Microsatellite genotyping

We dissected samples under a stereomicroscope to remove macroinvertebrates from the sponge tissue, and extracted DNA using the DNeasy® Blood and Tissue Kit (Qiagen). We genotyped the samples for 10 microsatellite loci, as described in Griffiths et al. (2019). Briefly, we amplified the loci in two multiplex PCR reactions (Griffiths et al. 2019) with the Type-it Microsatellite PCR Kit (Qiagen), using the following thermal cycling conditions: initial denaturation of 95 °C for 5 min, 28 cycles of 95 °C for 30 s, 60 °C (Multiplex A) or 63 °C (Multiplex B) for 90 s and 72 °C for 30 s, followed by a final extension of 60 °C for 30 min. Alleles were then sized using capillary electrophoresis on a DNA Analyzer 3730 (Thermo Fisher Scientific), and scored using Genemapper v3.7 (Thermo Fisher Scientific). All plates for PCR and genotyping contained positive and negative controls.

### Quality control and summary statistics

We tested linkage disequilibrium between loci using Genepop on the Web v4.2 (Raymond and Rousset 1995), and corrected significance for multiple tests (Benjamini and Yekutieli 2001; B–Y correction) using the *p.adjust* function in R 3.4.3 (R Core Team 2017). We calculated null allele frequencies in FreeNA (Chapuis and Estoup 2007) using the Expectation Maximisation algorithm (Dempster et al. 1977), and then conducted a post hoc analysis to determine the influence of null alleles on population differentiation estimation. This analysis was carried out by calculating global *F*_ST_ with and without correction for null alleles using the ENA method of Chapuis and Estoup (2007). Following this, the locus with the highest null allele frequency was removed, and *F*_ST_ with and without ENA correction was recalculated (sensu Chaves-Fonnegra et al. 2015). This was repeated sequentially until only one locus remained. This analysis revealed that the two loci with the highest null allele frequencies, Icam34 and Icam10, skewed uncorrected global *F*_ST_ estimates over 0.01 (Table S1). Consequently, in analyses where correction for null alleles could be implemented (*F*_ST_ and population-average inbreeding coefficient (*F*_IS_) calculations), the full ten-loci dataset was used. In all other analyses, Icam34 and Icam10 were excluded, yielding an eight-loci dataset.

We calculated probability of deviation from Hardy–Weinberg equilibrium (HWE), observed heterozygosity (*H*_O_) and Nei’s gene diversity (*H*_S_) (expected heterozygosity) for each sampling site using GenoDive v2.0b23 (Meirmans and Van Tienderen 2004). We calculated the average *F*_IS_ for each site corrected for null alleles in INEst v2.1 (Chybicki and Burczyk 2009), which uses a likelihood-based method to estimate *F*_IS_ and null allele frequencies simultaneously. We used the Interacting Multiple Model with 500,000 Markov chain Monte Carlo (MCMC) cycles and 50,000 burn-in cycles. We ran the model using all combinations of parameters for possible null allele causes (‘n’: null alleles; ‘b’: genotyping failure; ‘f’: inbreeding), and used Bayesian deviance information criterion (DIC) to infer which parameters contributed more to the observed data.

### Genetic diversity and bottlenecks

We assessed genetic diversity of the sites using ADZE (Szpiech et al. 2008), which uses a rarefaction method to calculate allelic richness and private allelic richness corrected for sample size variation. Gray’s Reef was excluded from this analysis due to its small sample size, and because the marker Icam3 did not amplify in any sample from this site. We used linear mixed models to test for differences in genetic diversity between sites, with site as a fixed effect, and locus as a random effect due to inter-marker variation in diversity (Soro et al. 2017; Maebe et al. 2018). We tested both rarefied allelic richness and *H*_S_ in separate models (eight-loci datasets), and ran the models using the R package ‘lme4’ (Bates et al. 2015). We used likelihood ratio tests to compare these models against their respective null models (i.e., excluding the fixed effect factor ‘site’). When significantly different, we ran a post hoc Tukey test to determine which sites significantly differed using the *glht* function in the R package ‘multcomp’ (Hothorn et al. 2008).

We tested for genetic signatures of bottlenecks at each sampling site by testing for significant heterozygosity excess in relation to allelic richness (Piry et al. 1999), as implemented in INEst v2.1. In bottleneck events, both heterozygosity and the number of alleles reduce; however, the allelic richness declines faster than heterozygosity, resulting in heterozygosity excess. We ran the two-phase mutation model with the proportion of multistep mutations set as 0.22, and the average multistep mutation size as 3.1, as recommended by Chybicki and Burczyk (2009). We ran the model using 100,000 coalescent simulations, and tested significance using the Wilcoxon signed-rank test, calculated based on 1,000,000 permutations.

### Population structure analysis

The traditional measure of subpopulation differentiation, *F*_ST_, can underestimate differentiation when variation in markers is high (Jost 2008). Because high numbers of alleles were present in a number of loci in our samples (up to 97 alleles per locus, Table S2), we also used Jost’s *D* to estimate population differentiation (Jost 2008). We calculated population pairwise *D* and *F*_ST_ in GenoDive using the eight-loci dataset (referred to as *D* and *F*_ST(8)_ hereafter), and tested significance using 50,000 permutations (with B–Y correction applied for multiple tests). We also calculated *F*_ST_ between population pairs for all ten loci with ENA correction for null alleles in FreeNA (referred to as *F*_ST(10)_ hereafter). We tested correlation between matrices of *F*_ST(8)_, *F*_ST(10)_ and *D*, using Mantel tests in the ‘ade4’ package in R with 9999 permutations.

To visualise genetic distance relationships among sites, we carried out principal coordinate analysis (PCoA) in GenAlEx 6.503 (Peakall and Smouse 2012) using standardised covariance matrices of population pairwise *D*.

We tested for the presence of genetic isolation-by-distance (IBD) patterns in the data by testing the correlation between pairwise matrices of linearised genetic distances (*F*_ST(10)_/1–*F*_ST(10)_ and *D*/1–*D*) and the logarithm of oceanographic distances with Mantel tests in ‘ade4’ in R, using 9999 permutations. We calculated least-cost oceanographic distances among sites (i.e., the shortest path, excluding landmasses) using ‘marmap’ in R (Pante and Simon-Bouhet 2013). We conducted these tests within the Lesser Antilles (Guadeloupe, Martinique, St. Lucia, St. Vincent, Bequia and Mayreau) and within the Florida Keys.

We used a spatially explicit Bayesian approach implemented in ‘Geneland’ v4.0.6 in R (Guillot et al. 2008) to identify the number of population clusters (*K*) and individual assignment probabilities to those clusters, using the eight-loci dataset. We first ran the model using the uncorrelated allele frequencies, spatial and null allele models with 1,000,000 iterations, 100 thinning and 500 burn-in. According to the authors’ advice, we set the maximum number of nuclei to 1320 (3 × sample size) and the maximum rate of the Poisson process to 440 (1 × sample size), and the spatial uncertainty on coordinates to 0.0005. We carried out ten independent runs of *K* from 1 to 19, and checked that clusters were consistent among runs. We then selected the run with the highest posterior probability to estimate allele frequencies and cluster locations to use subsequently in the admixture model. We ran the admixture model using the same parameters as above, and extracted the Q matrix of individual probability assignments to each cluster to build a bar plot in Distruct (Rosenberg 2004). We repeated the analysis on each identified cluster to test for the presence of substructure in a hierarchical clustering approach sensu Vaha et al. (2007), modifying the maximum number of nuclei and the maximum rate of the Poisson process according to the number of samples in the data subsets.

We carried out Discriminant Analysis of Principal Components (DAPC) (Jombart et al. 2010) using ‘adegenet’ (v2.0.1) (Jombart 2008) in R. This method uses Principal Component Analysis to transform the data, and then uses the retained principal components in Discriminant Analysis. This is effective in minimising within-group genetic variation and maximising between-group variation, and does not make assumptions regarding HWE in populations. The number of principal components retained varied among analyses; if too many are retained, the resulting membership probabilities can be unstable. We therefore retained the maximum number possible without compromising stability (displayed graphically in each DAPC plot). As before, we repeated the analysis for each multisite cluster identified.

## Results

### Summary statistics, HWE and *F*_IS_

All loci were in linkage equilibrium after B–Y correction (*p* > 0.05). Two identical multilocus genotypes (i.e., clones) were present in the dataset (both individuals from Turneffe Atoll), one of which was removed for subsequent analyses. Average null allele frequencies were high at many loci, overall ranging from <0.001 (Icam32) to 0.323 (Icam34) (Table S2). Loci were highly polymorphic, with the total number of alleles per locus ranging from 4 to 97 (Table S2).

There were significant departures from HWE (*p* < 0.05) at a number of loci and sites following the correction for multiple tests (Table S3). Null allele-corrected average *F*_IS_ values were all positive, ranging from 0.033 in Panama 2 to 0.450 in Bamboo Key, Florida (Table 2); however, posterior 95% probability intervals included zero for all sites but Bamboo Key. Model comparison using DIC values revealed that null alleles were important in affecting *F*_IS_ estimates in all sites, along with genotyping failure and/or inbreeding in some of the populations (Tables 2 and S4).

**Table 2:** Genetic diversity and average inbreeding coefficient in *Ircinia campana* per location.

Site name	Site code	*H*_O (8)_	*H*_E (8)_	Av*F*_IS_	Model	95% HPDI	AR	rAR ± SE	rPAR ± SE
Gray’s Reef	GR	0.325	0.528	0.037	nb	0–0.131	3.8	–	–
Long Key	LK	0.414	0.665	0.149	nb	0–0.339	8.6	4.319 ± 0.651	0.917 ± 0.289
Bamboo Key	BK	0.365	0.643	0.450	nfb	0.122–0.668	6.0	3.920 ± 0.556	0.675 ± 0.201
Kemp Channel	KC	0.305	0.578	0.099	n	0–0.291	7.3	3.635 ± 0.659	0.523 ± 0.219
Waltz Key	WK	0.317	0.557	0.226	nf	0–0.416	5.3	3.258 ± 0.552	0.237 ± 0.107
Boca Chica Channel	BC	0.377	0.65	0.148	nb	0–0.371	6.7	4.048 ± 0.597	0.750 ± 0.243
Lakes Passage	LP	0.365	0.644	0.092	nb	0–0.272	6.9	3.809 ± 0.556	0.758 ± 0.269
Turneffe Atoll	TA	0.397	0.603	0.047	nf	0–0.117	6.5	3.321 ± 0.446	0.533 ± 0.289
Sapodilla Cayes	SC	0.516	0.725	0.068	nfb	0–0.142	9.6	4.112 ± 0.384	1.215 ± 0.236
Panama 1	PAN1	0.509	0.634	0.077	nb	0–0.229	5.9	3.505 ± 0.541	0.554 ± 0.228
Panama 2	PAN2	0.565	0.695	0.033	nb	0–0.111	5.6	3.656 ± 0.413	0.569 ± 0.236
Panama 3	PAN3	0.466	0.661	0.057	nb	0–0.167	4.8	3.580 ± 0.503	0.602 ± 0.213
Mayreau	MAY	0.535	0.761	0.159	nfb	0–0.304	9.9	4.667 ± 0.589	1.229 ± 0.362
Bequia	BEQ	0.496	0.692	0.093	nb	0–0.237	11.8	4.627 ± 0.612	0.926 ± 0.274
St. Vincent	STV	0.457	0.654	0.034	nb	0–0.104	13.1	4.502 ± 0.662	1.135 ± 0.370
St. Lucia	STL	0.473	0.740	0.075	nb	0–0.195	13.0	4.585 ± 0.583	1.302 ± 0.432
Martinique 1	MAR1	0.495	0.687	0.102	n	0–0.251	7.8	4.380 ± 0.589	0.891 ± 0.286
Martinique 2	MAR2	0.502	0.627	0.048	n	0–0.135	8.7	4.027 ± 0.562	0.939 ± 0.281
Guadeloupe	GU	0.368	0.642	0.086	n	0–0.242	10.1	4.071 ± 0.621	1.212 ± 0.379

*H*_O(8)_ observed heterozygosity (over eight loci), *H*_E(8)_ expected heterozygosity (over eight loci), *AvF*_IS_ average inbreeding coefficient corrected for null alleles, *Model* model with lowest deviance information criteria for estimating the inbreeding coefficient in the presence of combinations of null alleles (n), inbreeding (f) and genotyping failure (b), *95% HPDI* 95% highest posterior density interval for Av*F*_IS_ calculation, *AR* average allelic richness, *rAR* *±* *SE* average rarefied allelic richness over ten loci (±standard error of the mean), maximum standardised sample size (max g) = 8, *rPAR* *±* *SE* average rarefied private allelic richness over ten loci (±standard error of the mean), maximum standardised sample size (max g) = 8.

### Genetic diversity and bottlenecks

*H*_O_ ranged from 0.305 (Kemp Channel) to 0.565 (Panama C) within sites, and *H*_S_ ranged from 0.528 (Gray’s Reef) to 0.761 (Mayreau) (Table 2). The linear mixed model showed that site had no significant effect on *H*_S_; however, the *p* value was marginal (*Χ*^2^ = 28.315, *p* = 0.057). Rarefied allelic richness per site ranged from 3.258 ± 0.552 SE (Waltz Key) to 4.667 ± 0.589 SE (Mayreau) (Table 2 and Fig. S1). Site had a significant effect on rarefied allelic richness according to the linear mixed model (*Χ*^2^ = 40.695, *p* = 0.001). However, post hoc Tukey tests revealed that only three pairs of sites were significantly different: allelic richness was significantly higher at St. Lucia than at Turneffe Atoll (*z* = −4.494, *p* = 0.049), at Mayreau than at Turneffe Atoll (*z* = −3.859, *p* = 0.013) and at Mayreau than at Waltz Key (*z* = −3.738, *p* = 0.022). All other site-by-site comparisons were not significant (*p* > 0.05), although three comparisons were close to significance (higher allelic richness at St. Lucia than at Waltz Key: *z* = −3.373, *p* = 0.072, at Bequia than at Turneffe Atoll: *z* = −3.421, *p* = 0.062 and at Bequia than at Waltz Key: *z* = −3.300, *p* = 0.083). Rarefied private allelic richness ranged from 0.237 ± 0.107 (Waltz Key) to 1.302 ± 0.432 (St. Lucia) (Table 2 and Fig. S1).

Significant heterozygosity excess with respect to allelic richness was detected only at Bamboo Key (*p* = 0.014), indicative of a genetic bottleneck.

### Population structure

Our analyses showed strong population structure across the region, with *F*_ST(10)_ reaching 0.233 (between Gray’s Reef and Panama 1) and *D* reaching 0.598 (between Gray’s Reef and Sapodilla Cayes) (Tables S5 and S6). The genetic distance measures were strongly correlated (*F*_ST(10)_ and *F*_ST(8)_: *r* = 0.921, *p* < 0.001; *F*_ST(8)_ and *D*: *r* = 0.908, *p* < 0.001; *F*_ST(10)_ and *D*: *r* = 0.825, *p* < 0.001).

**Fig. 2:**
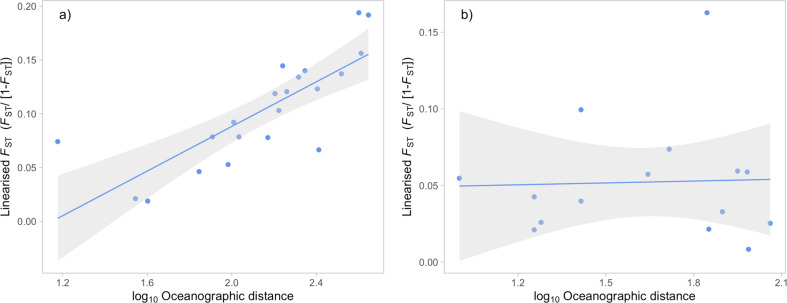
Genetic isolation by distance in *Ircinia campana*. Distances displayed are site pairwise linearised *F*_ST_ and logarithm of oceanographic distances. (**a**) Sites within the Lesser Antilles (**b**) Sites within the Florida Keys.

IBD was significant within the Lesser Antilles subset (pairwise distances 15–443 km; *F*_ST(10)_ *r* = 0.793, *p* = 0.001; *D*: *r* = 0.688, *p* < 0.001) (Fig. 2a) but not within the Florida subset (pairwise distances 10–115 km; *F*_ST(10)_: *p* = 0.483; *D*: *p* = 0.476) (Fig. 2b).

**Fig. 3:**
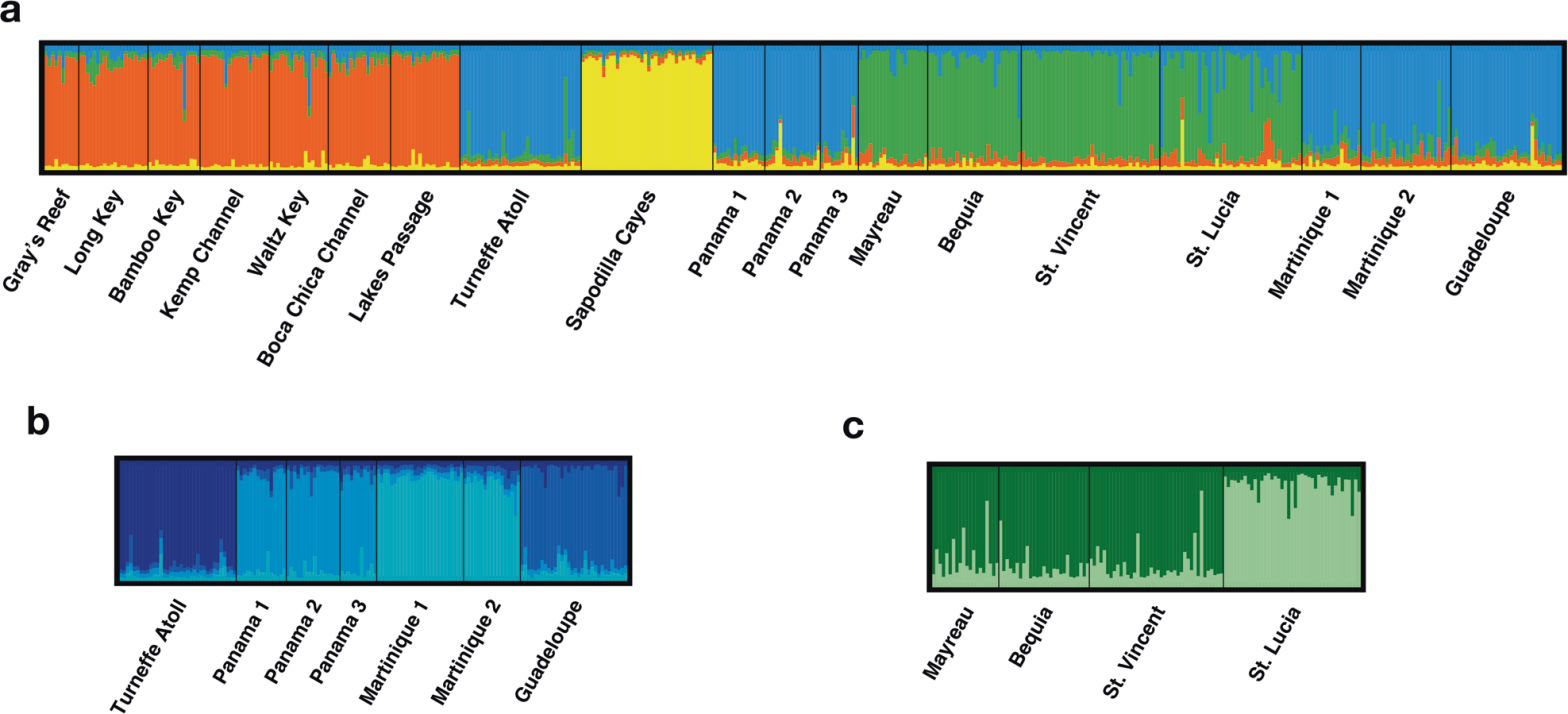
Geneland plot showing genetic clusters and individual admixture proportions in *Ircinia campana*. Each bar represents an individual; colours represent genetic cluster identity, and bar heights represent inferred membership proportions to genetic clusters. The analysis was firstly carried out over all sites, showing *K* = 4 genetic clusters (**a**); separate analyses were subsequently conducted on multisite clusters identified therein (**b**, **c**), showing *K* = 4 (**b**) and *K* = 2 (**c**) genetic clusters. Analysis on the USA cluster yielded *K* = 1 (data not shown).

Geneland analysis of the whole dataset identified four genetic clusters (Fig. 3a), with further population structure identified on subsequent hierarchical analysis of the original clusters. Clusters were composed as follows: (1) all USA sites, (2) the southern Lesser Antilles sites (St. Lucia, St. Vincent, Mayreau and Bequia), (3) the Panama, Turneffe Atoll, Guadeloupe and Martinique sites, and (4) the Sapodilla Caye site in southern Belize alone. Subsequent analysis of multisite clusters identified further substructure between St. Lucia and St. Vincent, Mayreau and Bequia (Fig. 3b), and between Turneffe Atoll, Panama, Martinique and Guadeloupe (Fig. 3c), giving a total of eight clusters over all analyses. The USA sites remained a single cluster.

**Fig. 4:**
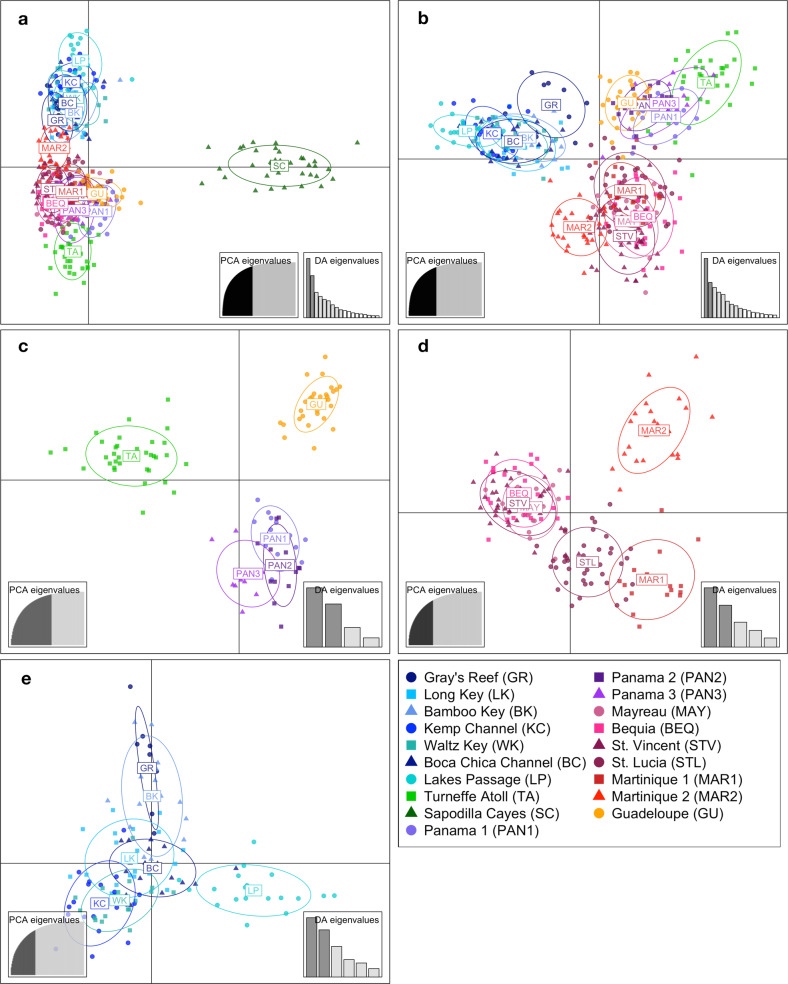
Discriminant analyses of principal components (DAPC) of *Ircinia campana*. Individual points represent genotyped individuals; inertia ellipses summarise the point cloud for each site. Sequential analyses were carried out in a hierarchical approach by repeating analyses on clusters detected (**a**–**e**). Insets show the proportion of principal component eigenvalues and discriminant analysis eigenvalues retained.

The DAPC showed a clear separation of the Sapodilla Cayes from all other sites upon analysis of the full dataset (Fig. 4a). In the subsequent analysis excluding the Sapodilla Cayes, sites formed three clusters (Fig. 4b), comprising (1) all USA sites, (2) St. Vincent, Mayreau, Bequia, St. Lucia and Martinique and (3) Panama, Turneffe Atoll and Guadeloupe (Fig. 4b). DAPC analyses conducted on these clusters revealed further substructure (Fig. 4c–e): Panama, Guadeloupe and Turneffe Atoll separated into individual groups (Fig. 4c); Bequia, Mayreau and St. Vincent clustered together, while St. Lucia, Martinique 1 and Martinique 2 formed separate but closely located clusters (Fig. 4d). In common with other analyses, the USA sites did not separate into multiple clusters, although Lakes Passage was the most differentiated (Fig. 4e).

**Fig. 5:**
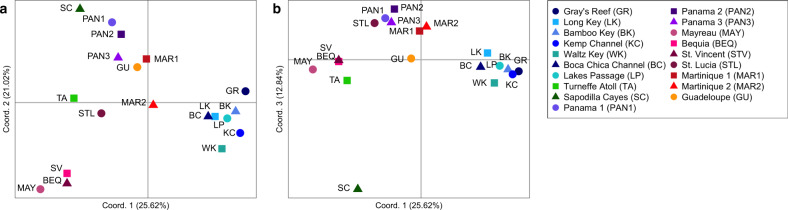
Principal coordinate analysis (PCoA) of Jost’s *D* genetic distance among *Ircinia campana* sampling sites. Genetic distances calculated using eight microatellite loci dataset. (**a**) Axis 1 vs axis 2 (**b**) axis 1 vs axis 3.

PCoA plots showed some regional-scale clustering patterns consistent with the Geneland and DAPC analyses (Fig. 5). The first axis represented 25.62% of the variation in *D* among sites, the second axis represented 21.02% and the third axis represented 12.48%, giving a total of 59.12% over the three axes. The first axis separated the USA sites from the rest of the sites, while the second axis clearly separated a St. Vincent, Mayreau and Bequia cluster (Fig. 5a). The third axis separated the Sapodilla Cayes from all other sites (Fig. 5b).

## Discussion

### Departures from HWE and null alleles

We observed significant deviation from HWE at many loci and sites. There are many potential biological causes of this, including inbreeding, which increases population homozygosity. In sponges, inbreeding could result from low dispersal of planktonic sperm and larvae, which can lead to philopatry and higher incidences of non-random mating.

Another possible cause of HWE departure is genetic structure within a population, or a set of samples, known as the Wahlund effect. In a single geographical site, this could be caused by stochasticity, variation in selective pressures and changes in ocean circulation that alter dispersal patterns over time (Tesson et al. 2014). Although samples were collected over a 3-year period, we do not believe that this caused temporal structure in our dataset, as sponges are sessile and slow-growing. The high polymorphism of the microsatellite loci used in this study may have also contributed to HWE deviation, as HWE tests are very sensitive to individuals homozygous for rare alleles (Morin et al. 2009).

Deviation from HWE can also be caused by null alleles, which were indeed found at the majority of loci. Null alleles increase estimates of homozygosity, subsequently raising *F*_IS_ values. In addition, null alleles can artificially increase *F*_ST_ values and estimates of population differentiation (Chapuis and Estoup 2007). To mitigate these effects as far as possible, we corrected *F*_ST_ values and population-average *F*_IS_ values for null alleles, we used the null allele model in Geneland and we excluded the loci that contributed substantially to *F*_ST_ skew from other analyses.

Both HWE deviations and null alleles are common in sponge microsatellite studies (Dailianis et al. 2011; Chaves-Fonnegra et al. 2015; Pérez-Portela et al. 2015; Giles et al. 2015; Guardiola et al. 2016; Richards et al. 2016), suggesting that common life history trends could contribute to these observations. High *F*_IS_ and HWE departures are common among marine invertebrates with free-spawned planktonic sperm. Addison and Hart (2005) proposed that this could be associated with higher levels of null alleles, due to higher numbers of cell cycles for sperm production causing increased mutation rates (though sperm production varies interspecifically, and is unknown for *I. campana*).

### Regional-scale population structure

Our results show the presence of strong regional population structure in *I. campana* in the Greater Caribbean, with a total of eight to nine genetic clusters identified, indicating highly differentiated populations across the region. This agrees with studies of the Caribbean sponges *C. delitrix* (using microsatellites; Chaves-Fonnegra et al. 2015) and *C. vaginalis* (using mtDNA and nuclear genes; DeBiasse et al. 2016), supporting that the short-lived nature of sponge larvae constrains dispersal, and is an important driver of population structure across the phylum.

The *I. campana* population in the Sapodilla Cayes was genetically distinct from all other sites according to the DAPC and Geneland analyses. In addition, *D* values were almost always higher for all population pairwise comparisons involving this site than for any other population pairs. The Sapodilla Cayes is situated in the south of the Mesoamerican Barrier Reef System (MBRS), an area subject to highly retentive oceanographic conditions (Martínez et al. 2019): a weak southward-flowing coastal current and the anticlockwise Gulf of Honduras Gyre (Ezer et al. 2005; Carrillo et al. 2015), contrasting with the northerly flowing Yucatan Current in the north MBRS. Furthermore, river discharge into the Gulf of Honduras may form an additional barrier to dispersal for stenohaline marine larvae, such as *Ircinia* spp. (Soto et al. 2009). Genetic studies show population differentiation between the north and south MBRS in the corals *Montastrea annularis* (Foster et al. 2012) and *Orbicella faveolata* (Rippe et al. 2017), as well as the neon goby *Elacatinus lori* (D’Aloia et al. 2017). Genetic evidence also suggests that the lobster *Panulirus argus* experiences higher self-recruitment in the south (Truelove et al. 2014). In addition, Muhling et al. (2013) found distinct larval fish assemblages in the north and south MBRS. The breadth of taxa that are affected by this divide, with their varied life histories, supports that ocean circulation is driving differentiation in this area.

The high level of genetic differentiation found at the Sapodilla Cayes in *I. campana* may be a case of cryptic speciation, as suggested for the neon goby *E. lori* in the same region (D’Aloia et al. 2017). Cryptic species in sponges are common due to absences in morphological variation caused by a lack of complex morphological traits, as well as phenotypic plasticity or convergent evolution resulting in similar morphologies (Sole-Cava et al. 1991; Xavier et al. 2010). However, further studies using phylogenetically informative markers are needed to explore this possibility in *I. campana*.

Excluding the Sapodilla Cayes, the remaining sites split into three main clusters in both the DAPC and Geneland analyses prior to further hierarchical analyses. The composition of clusters was consistent among analyses, except for the Martinique sites, which clustered with Turneffe Atoll, Panama and Guadeloupe in the Geneland analysis, and with Bequia, Mayreau, St Vincent and St. Lucia in the DAPC. However, cluster-based models are known to not perform as well when genetic variation does not fall into discrete groups, but follow a strong IBD pattern, as observed in the Lesser Antilles (Guillot et al. 2005).

The cluster formed by Turneffe Atoll, Panama and Guadeloupe (and Martinique in the Geneland analysis) is unexpected, given the genetic and geographic distances among these locations, and the absence of specific water circulation patterns that might cause such a grouping. However, when analyses were repeated including only these sites, the sites divided into three separate clusters. This demonstrates the utility of repeating analyses on multisite clusters to uncover further patterns of population structure that are not revealed in the overall dataset analysis (Janes et al. 2017). The preliminary grouping of these sites may be partially due to size homoplasy in the microsatellites, which occurs when different alleles are identical lengths, and are consequently scored as the same allele—potentially creating spurious links (Estoup et al. 2002).

The sites in the United States formed a single cluster, comprising the Florida Keys sites and Gray’s Reef National Marine Sanctuary, despite a distance of ~770 km separating these areas, and the moderate *F*_ST_ and *D* values. Although direct larval transport between the areas is very unlikely, indirect connectivity could be maintained via larval transportation in the Florida Current, coupled with ‘stepping stones' of suitable coastal habitat harbouring intermediate populations along the south eastern coast of mainland USA. The USA cluster is probably distinct from the other Caribbean sites sampled due to distance rather than any particular oceanographic barrier; further fine-scale sampling of sites in the Greater Antilles and Gulf of Mexico would be required to explore this further.

### Population structure at smaller spatial scales

The Mantel test indicated strong IBD within the Lesser Antilles (where distances between sites ranged 15–443 km). IBD is driven by distance-limited dispersal (Wright 1943; Aguillon et al. 2017), which is consistent with the short planktonic duration common for lecithotrophic sponge larvae. IBD patterns were also observed in the coral *A. palmata* in this area (Japaud et al. 2019), and have been found in other sponges at similar spatial scales elsewhere (Bell et al. 2014; Pérez-Portela et al. 2015; Riesgo et al. 2016). However, many sponge studies have found IBD to be absent or very weak, with oceanographic conditions forming better predictors of population structure (Giles et al. 2015; Taboada et al. 2018; Riesgo et al. 2019). This suggests that when oceanographic barriers are absent, limited larval dispersal in sponges can cause distance-decay relationships in genetic structuring.

In contrast, genetic IBD was not significant in the Florida Keys archipelago, where distances between sites ranged 10–115 km. Pairwise genetic differentiation between sites in Florida was relatively low (null allele-corrected *F*_ST_ ranged between 0.006 and 0.072), and the sites formed a single cluster in Geneland, PCoA and DAPC analyses. This suggests that larvae disperse across the area with sufficient regularity to maintain gene flow (although genetic similarity does not exclude the possibility of recent divergence). This result is interesting given the short larval duration and low dispersal capacity predicted for sponges, but concurs with other studies that show higher-than-expected levels of connectivity and dispersal (Chaves-Fonnegra et al. 2015; de Bakker et al. 2016). In other sponges, stronger genetic structure has been observed (e.g., Riesgo et al. 2019), most strikingly on spatial scales of centimetres to tens of metres in *Crambe crambe* and *Scopalina lophyropoda* (Calderón et al. 2007; Blanquer et al. 2009). This variation could be due to larval characteristics (Uriz et al. 2008); compared with other sponges, Irciniidae larvae are relatively strong swimmers with larger lipid stores (Ereskovsky and Tokina 2004; Mariani et al. 2006), theoretically aiding dispersal.

Although the Florida Keys formed a single genetic cluster, 8 out of 15 pairs of sites showed significant but low genetic differentiation, with no site consistently emerging as different from the rest—a common pattern in marine systems termed ‘chaotic genetic patchiness’ (Johnson and Black 1982). Chaotic genetic patchiness with weak or no IBD has also been found in other sponges across the Florida reef tract (DeBiasse et al. 2010; Chaves-Fonnegra et al. 2015; Richards et al. 2016), and in a co-occurring sponge, *S. vesparium* (Griffiths et al. 2020), which shared many sampling sites with this study.

Chaotic genetic patchiness can potentially result from a number of different processes (Eldon et al. 2016). One proposed cause is the random survival of larval cohorts due to stochastic oceanographic conditions, found in species with high fecundity and high larval mortality, termed ‘sweepstakes reproductive success’ (Hedgecock and Pudovkin 2011; Jolly et al. 2014). Another possible cause of temporal variation in recruitment and dispersal dynamics could be variability in local hydrodynamics across time (Schunter et al. 2019). Water circulation is highly variable among shallow, nearshore areas in Florida due to the predominance of wind-driven currents and storms regularly altering bathymetry. Asynchronicity in reproduction within populations could be a further component of temporal variability in recruitment dynamics (Eldon et al. 2016). Another possible cause is differential post-settlement selection (Norderhaug et al. 2016); in *I. campana*, this could result from high selection pressure exerted by cyanobacterial blooms (Butler et al. 1995) or disease (Maldonado et al. 2010). Indeed, we found evidence of a genetic bottleneck at Bamboo Key following a known mass mortality caused by a cyanobacterial bloom (see section below).

In Martinique, genetic differentiation was higher than expected between the two sampling sites (*F*_ST_ = 0.063) considering their proximity (15 km). In addition, the DAPC and PCoA analyses highlighted genetic separation between the sites. Diamond Rock (MAR2) is a small island located 3 km off the Martinique coast. Both the island and the channel separating it from the mainland are locally known to experience strong currents (pers. comm. G. Tollu), and early modelling of water circulation suggests that the area may be influenced by a small gyre off the southern coast of Martinique (Lazure et al. 1996), which could reduce connectivity between the sites. However, more in-depth work on local water movement patterns is needed to further interpret the cause of this genetic differentiation.

### Genetic diversity and bottlenecks

We found evidence of a genetic bottleneck in Bamboo Key, where previous mass mortalities were observed as part of widespread, reoccurring mass mortalities in sponge communities across the Florida Keys associated with cyanobacterial blooms (Butler et al. 1995; Stevely et al. 2010). This prompts concern for the population’s resilience and adaptability to future stressors (Wernberg et al. 2018). Bottleneck signatures have also been found in other sponges in Mediterranean sites due to overharvesting (Pérez-Portela et al. 2015) and disease (Riesgo et al. 2016), underscoring the risks to genetic diversity levels for sponge populations that undergo significant declines. However, as null alleles can affect estimates of both heterozygosity and allelic richness, the possibility of a false positive at Bamboo Key cannot be excluded, given the presence of null alleles in our dataset.

Past mortalities also occurred in another of our sampling sites (Long Key in Florida), but a bottleneck signature was not detected here. This could be because any loss in genetic diversity was rapidly regained through recruitment and gene flow from other areas. Indeed, the Long Key site is dominated by long-shore currents, whereas the Bamboo Key site sits within a bay subject to a local gyre. Alternatively, it is possible that the Long Key result is a false negative, as heterozygosity excess tests are sensitive to mutation model selection and sample-size limitations (Garza and Williamson 2001; Peery et al. 2012). However, we found that genetic diversity was generally high in *I. campana*, with similar *H*_S_ ranges to other sponges (Chaves-Fonnegra et al. 2015; Giles et al. 2015; Richards et al. 2016; Riesgo et al. 2019); furthermore, allelic richness and *H*_S_ in Long Key were not significantly different from other sites.

Rapid population declines have not been reported in any of our other study sites, nor were significant bottleneck signatures detected. In addition, genetic diversity was largely similar across sites, with only Waltz Key and Turneffe Atoll showing significantly lower allelic richness than some Lesser Antilles sites (Mayreau, St. Lucia and Bequia). However, we did observe a disease-like condition in individuals harbouring necrosis of various stages in Guadeloupe and Bequia during sampling for this study, and on other occasions in Martinique (pers. obs. T. Pérez and S. Griffiths). Indeed, disease outbreaks and mass mortalities have been reported multiple times in *Ircinia*, often linked with higher temperatures (Perez et al. 2000; Maldonado et al. 2010; Stabili et al. 2012; Riesgo et al. 2016). Given this vulnerability, and the bottleneck signature found in Bamboo Key, continued genetic monitoring of *I. campana* would be prudent to ensure that bottlenecks are accurately identified for effective management of the species (Schwartz et al. 2007).

### Conservation implications

Understanding the scale and magnitude of connectivity among populations is important for the management and conservation of marine ecosystems (Almany et al. 2009). Firstly, our results contribute to the increasing evidence suggesting that, for a number of taxa, the Sapodilla Cayes experiences low connectivity with other areas, and relies on high self-recruitment. This supports the current protection status for this area (the Sapodilla Cayes Marine Reserve), which is important to bolster the resilience of the population at this location, and to protect its unique genetic diversity. This study also implies that this population may form a separate management unit from other Belizean Marine Protected Areas (MPA); however, more extensive sampling of Belizean MPAs would be required to explore this further. More generally, our results tentatively suggest that to form connected MPAs for this species, protected areas would need to be situated within ~50–100 km of each other, where suitable habitat exists and oceanographic barriers are absent. This would allow sufficient spillover of larvae to non-protected areas, and would maintain connectivity between protected areas.

Our results also provide insight for management of mass mortality-affected sites in Florida. Restoration through fragmenting and transplanting healthy sponges has been successfully used to repopulate barren areas (Butler et al. 2016). Our results suggest that gene flow occurs over the length of the Keys, indicating that donor sponges may be sourced from any area of the Keys without risk of outbreeding depression. Our results also indicate that connectivity over the area is unpredictable; therefore, restoration is supported as an important strategy to ensure rapid repopulation of mass mortality-affected areas. Genetic diversity is naturally high in *I. campana* populations; this should be maintained in restored sites through the use of a large number of donor sponges, rather than extensive fragmentation of few sponges.

## Supplementary information

Supplementary Information

## Data Availability

Microsatellite genotype data are available at 10.5061/dryad.gtht76hh7.
