# Supplementary material for: Oceanographic features and limited dispersal shape the population genetic structure of the vase sponge Ircinia campana in the Greater Caribbean
Source: Heredity (Edinb). 2020 Jul 22;126(1):63–76. doi: 10.1038/s41437-020-0344-6 (PMC7852562; doi:10.1038/s41437-020-0344-6)
Supplement: Supplementary file 1 — Supplementary Information [file 41437_2020_344_MOESM1_ESM.docx]

**Supplementary Information: Population genetic structure of the vase sponge *Ircinia campana* in the Greater Caribbean is shaped by oceanographic features and limited dispersal**

S.M. Griffiths, M.J. Butler IV, D.C. Behringer, T. Pérez & R.F. Preziosi

| **Number of loci 🡪** | **1** | **2** | **3** | **4** | **5** | **6** | **7** | **8** | **9** | **10** |
| --- | --- | --- | --- | --- | --- | --- | --- | --- | --- | --- |
| **Average null allele frequency** | 0.000 | 0.042 | 0.059 | 0.069 | 0.097 | 0.200 | 0.236 | 0.239 | 0.250 | 0.323 |
| **Global *F*_ST_ without ENA** | 0.035 | 0.126 | 0.162 | 0.127 | 0.132 | 0.152 | 0.141 | 0.128 | 0.134 | 0.141 |
| **Global *F*_ST_ with ENA** | 0.035 | 0.140 | 0.162 | 0.127 | 0.130 | 0.143 | 0.133 | 0.120 | 0.123 | 0.126 |
| **Difference in *F*_ST_ with and without ENA** | 0.000 | -0.014 | 0.000 | 0.000 | 0.002 | 0.009 | 0.008 | 0.008 | 0.011 | 0.015 |

**Table S1: Cumulative average null allele frequency and global *F*_ST_ calculated with and without ENA correction for null alleles. Beginning with all ten loci, global *F*_ST_ was calculated with and without ENA correction for null alleles (Chapuis and Estoup 2007). The locus with the highest null allele frequency was then removed from the analysis, after which the global *F*_ST_ statistics were recalculated. This process was repeated sequentially until one locus remained.**

**Table S2: Number of alleles and null allele frequency per locus**

|  | **Icam 3** | **Icam 4** | **Icam 10** | **Icam 18** | **Icam 23** | **Icam 24** | **Icam 26** | **Icam 31** | **Icam 32** | **Icam34** |
| --- | --- | --- | --- | --- | --- | --- | --- | --- | --- | --- |
| **Number of alleles** | 32 | 19 | 19 | 96 | 97 | 83 | 21 | 21 | 4 | 58 |
| **Null allele frequency** | 0.097 | 0.042 | 0.250 | 0.069 | 0.236 | 0.239 | 0.200 | 0.059 | <0.001 | 0.323 |

**Table S3: Inbreeding coefficient (*F*_IS_) values per locus per site for *Ircinia campana* (without null allele correction).**

|  | **Icam3** | **Icam4** | **Icam10** | **Icam18** | **Icam23** | **Icam24** | **Icam26** | **Icam31** | **Icam32** | **Icam34** |
| --- | --- | --- | --- | --- | --- | --- | --- | --- | --- | --- |
| **GR** | - | - | **1.000** | -0.118 | 0.009 | 1 | 1 | 0.294 | -0.125 | 1.000 |
| **LK** | -0.120 | 0.000 | **0.92** | **0.268** | **0.420** | **0.927** | **0.543** | 0.219 | -0.086 | **0.906** |
| **BK** | -0.103 | - | **0.82** | **0.412** | **0.699** | 0.543 | **0.585** | **0.755** | -0.250 | **0.797** |
| **KC** | 0.631 | - | **0.781** | 0.049 | **0.579** | **0.657** | **0.592** | **0.437** | - | 1.000 |
| **WK** | 0.141 | - | **0.783** | **0.306** | **0.611** | **0.480** | **0.685** | 0.432 | -0.067 | 1.000 |
| **BC** | 0.489 | - | **1.000** | **0.226** | 0.327 | **0.740** | **0.676** | 0.088 | -0.062 | **0.705** |
| **LP** | 0.282 | - | **0.591** | 0.166 | **0.350** | **0.745** | **0.626** | **0.525** | -0.056 | **0.793** |
| **TA** | -0.137 | 0.304 | **0.919** | 0.148 | **0.846** | **0.432** | **0.704** | 0.148 | -0.115 | **1.000** |
| **SC** | 0.166 | 0.191 | **0.599** | **0.363** | 0.131 | **0.619** | **0.719** | -0.004 | -0.007 | **0.734** |
| **PAN1** | 0.084 | -0.054 | 0.649 | -0.090 | **0.696** | **0.423** | 0.347 | -0.037 | -0.217 | **0.718** |
| **PAN2** | 0.182 | -0.119 | 0.672 | 0.225 | **0.570** | 0.066 | 0.350 | 0.160 | -0.340 | **0.629** |
| **PAN3** | -0.155 | -0.071 | - | 0.030 | **1** | **0.567** | 0.306 | 0.125 | -0.111 | **0.479** |
| **MAY** | **0.342** | **0.398** | **0.688** | 0.135 | **0.584** | **0.482** | **0.451** | 0.026 | -0.295 | **0.692** |
| **BEQ** | **0.437** | 0.116 | **0.575** | **0.154** | **0.694** | **0.277** | 0.343 | -0.025 | -0.163 | **0.643** |
| **STV** | **0.651** | 0.198 | **0.299** | -0.008 | **0.535** | **0.432** | **0.460** | -0.124 | -0.183 | **0.775** |
| **STL** | 0.170 | **0.696** | **0.672** | **0.132** | **0.480** | **0.489** | **0.619** | 0.227 | -0.048 | **0.953** |
| **MAR1** | 0.092 | -0.043 | **0.281** | 0.196 | **0.310** | **0.629** | -0.295 | -0.007 | -0.103 | **1.000** |
| **MAR2** | 0.094 | -0.020 | **0.660** | **0.274** | **0.640** | **0.380** | **0.516** | -0.036 | -0.064 | **0.811** |
| **GU** | 0.536 | 0.659 | **0.649** | 0.046 | **0.726** | **0.561** | **0.591** | -0.008 | -0.016 | **0.694** |

Significant p values after Benjamini-Yekutieli correction for multiple tests highlighted in bold (p < 0.05)

| **Site** | **Model** | | | | | |
| --- | --- | --- | --- | --- | --- | --- |
|  | **nfb** | **nf** | **nb** | **bf** | **n** | **b** |
| **GR** | 291.989 | 293.466 | **290.59** | - | 292.542 | - |
| **LK** | 1067.314 | 1090.907 | **1065.222** | 1097.398 | 1081.915 | 1227.870 |
| **BK** | **664.063** | 675.292 | 666.018 | 666.089 | 675.697 | 762.715 |
| **KC** | 926.179 | 925.259 | 926.460 | 946.704 | **925.147** | 1056.005 |
| **WK** | 672.205 | **672.110** | 676.515 | 684.412 | 677.906 | 738.229 |
| **BC** | 857.693 | 862.754 | **857.453** | 867.695 | 862.041 | 957.969 |
| **LP** | 972.074 | 971.871 | **971.097** | 986.096 | 971.113 | 1056.805 |
| **TA** | 1485.159 | **1483.566** | 1488.071 | 1551.031 | 1488.037 | 1683.175 |
| **SC** | **2141.152** | 2150.320 | 2146.490 | 2181.035 | 2154.532 | 2314.246 |
| **PAN1** | 487.430 | 496.328 | **486.003** | 497.951 | 495.728 | 515.232 |
| **PAN2** | 703.262 | 713.151 | **701.930** | 721.475 | 711.647 | 731.595 |
| **PAN3** | 806.103 | 813.974 | **805.861** | 814.440 | 813.670 | 829.725 |
| **MAY** | **1246.373** | 1247.470 | 1249.000 | 1256.874 | 1248.051 | 1338.043 |
| **BEQ** | 1720.730 | 1730.438 | **1718.836** | 1739.943 | 1728.436 | 1852.124 |
| **STV** | 2389.176 | 2428.908 | **2387.637** | 2448.622 | 2427.530 | 2635.384 |
| **STL** | 2596.634 | 2605.839 | **2594.866** | 2644.272 | 2603.508 | 2894.867 |
| **MAR1** | 1020.657 | 1019.150 | 1020.065 | 1044.973 | **1019.061** | 1120.244 |
| **MAR2** | 1406.457 | 1406.817 | 1405.281 | 1438.552 | **1405.035** | 1504.624 |
| **GU** | 1677.984 | 1674.430 | 1676.310 | 1705.252 | **1673.548** | 1900.984 |

**Table S4: Deviance information criterion (DIC) using various combinations of parameters in inbreeding coefficient (*F*_IS_) estimation models in *Ircinia campana*.**

n: null alleles; f: inbreeding; b: genotyping error. Lowest DIC values are highlighted in bold; these correspond to the best-fitting model for the data.

**Table S5 – Pairwise *F*_ST_ (below diagonal, shaded) and Jost’s *D* (above diagonal) calculated from 8 loci dataset**

|  | **GR** | **LK** | **BK** | **KC** | **WK** | **BC** | **LP** | **TA** | **SC** | **PAN1** | **PAN2** | **PAN3** | **MAY** | **BEQ** | **STV** | **STL** | **MAR1** | **MAR2** | **GU** |
| --- | --- | --- | --- | --- | --- | --- | --- | --- | --- | --- | --- | --- | --- | --- | --- | --- | --- | --- | --- |
| **GR** | - | **0.202** | 0.086 | **0.201** | **0.332** | **0.197** | **0.236** | **0.432** | **0.598** | **0.427** | **0.377** | **0.276** | **0.507** | **0.437** | **0.470** | **0.390** | **0.277** | **0.304** | **0.303** |
| **LK** | **0.112** | - | 0.057 | **0.036** | **0.066** | 0.009 | 0.032 | **0.322** | **0.477** | **0.237** | **0.185** | **0.186** | **0.324** | **0.206** | **0.258** | **0.179** | **0.124** | **0.105** | **0.189** |
| **BK** | 0.059 | 0.027 | - | **0.116** | **0.167** | 0.065 | **0.111** | **0.379** | **0.509** | **0.329** | **0.261** | **0.230** | **0.390** | **0.291** | **0.324** | **0.291** | **0.226** | **0.172** | **0.229** |
| **KC** | **0.138** | 0.022 | **0.069** | - | 0.012 | **0.075** | **0.080** | **0.378** | **0.526** | **0.328** | **0.314** | **0.276** | **0.403** | **0.281** | **0.295** | **0.266** | **0.231** | **0.150** | **0.261** |
| **WK** | **0.221** | **0.039** | **0.100** | 0.008 | - | **0.100** | **0.112** | **0.376** | **0.548** | **0.343** | **0.349** | **0.309** | **0.385** | **0.250** | **0.274** | **0.330** | **0.295** | **0.209** | **0.307** |
| **BC** | **0.118** | 0.005 | 0.032 | **0.046** | **0.062** | - | 0.030 | **0.282** | **0.446** | **0.254** | **0.223** | **0.180** | **0.316** | **0.212** | **0.239** | **0.243** | **0.148** | **0.124** | **0.166** |
| **LP** | **0.139** | 0.016 | **0.056** | **0.048** | 0.068 | **0.016** | - | **0.369** | **0.501** | **0.298** | **0.272** | **0.251** | **0.397** | **0.269** | **0.299** | **0.250** | **0.181** | **0.159** | **0.243** |
| **TA** | **0.247** | **0.16** | **0.191** | **0.207** | **0.21** | **0.148** | **0.185** | - | **0.463** | **0.294** | **0.341** | **0.275** | **0.337** | **0.231** | **0.261** | **0.322** | **0.273** | **0.287** | **0.247** |
| **SC** | **0.243** | **0.172** | **0.185** | **0.213** | **0.225** | **0.17** | **0.185** | **0.19** | - | **0.410** | **0.431** | **0.421** | **0.558** | **0.503** | **0.559** | **0.501** | **0.413** | **0.492** | **0.406** |
| **PAN1** | **0.237** | **0.112** | **0.157** | **0.176** | **0.188** | **0.124** | **0.144** | **0.154** | **0.156** | - | **0.091** | **0.085** | **0.465** | **0.332** | **0.360** | **0.256** | **0.149** | **0.212** | **0.250** |
| **PAN2** | **0.194** | **0.080** | **0.115** | **0.153** | **0.172** | **0.099** | **0.119** | **0.159** | **0.148** | **0.044** | - | 0.064 | **0.399** | **0.293** | **0.356** | **0.218** | **0.155** | **0.182** | **0.208** |
| **PAN3** | **0.164** | **0.086** | **0.110** | **0.148** | **0.167** | **0.087** | **0.118** | **0.142** | **0.153** | **0.045** | 0.030 | - | **0.317** | **0.225** | **0.257** | **0.245** | **0.195** | **0.145** | **0.193** |
| **MAY** | **0.205** | **0.115** | **0.138** | **0.166** | **0.162** | **0.116** | **0.145** | **0.141** | **0.163** | **0.165** | **0.129** | **0.111** | - | **0.060** | **0.104** | **0.212** | **0.382** | **0.296** | **0.381** |
| **BEQ** | **0.211** | **0.089** | **0.124** | **0.137** | **0.126** | **0.094** | **0.118** | **0.113** | **0.171** | **0.142** | **0.114** | **0.096** | **0.022** | - | **0.032** | **0.192** | **0.269** | **0.194** | **0.248** |
| **STV** | **0.239** | **0.118** | **0.147** | **0.152** | **0.146** | **0.114** | **0.14** | **0.134** | **0.202** | **0.165** | **0.148** | **0.118** | **0.043** | **0.015** | - | **0.234** | **0.311** | **0.219** | **0.315** |
| **STL** | **0.165** | **0.069** | **0.109** | **0.115** | **0.141** | **0.094** | **0.097** | **0.135** | **0.154** | **0.099** | **0.077** | **0.090** | **0.066** | **0.070** | **0.092** | - | **0.117** | **0.160** | **0.251** |
| **MAR1** | **0.183** | **0.056** | **0.100** | **0.118** | **0.150** | **0.069** | **0.084** | **0.134** | **0.145** | **0.07** | **0.065** | **0.085** | **0.127** | **0.108** | **0.134** | **0.043** | - | **0.125** | **0.129** |
| **MAR2** | **0.147** | **0.055** | **0.091** | **0.089** | **0.123** | **0.067** | **0.085** | **0.153** | **0.189** | **0.111** | **0.087** | **0.076** | **0.118** | **0.091** | **0.109** | **0.067** | **0.063** | - | **0.168** |
| **GU** | **0.171** | **0.092** | **0.111** | **0.141** | **0.164** | **0.084** | **0.119** | **0.131** | **0.159** | **0.123** | **0.095** | **0.094** | **0.143** | **0.111** | **0.147** | **0.100** | **0.062** | **0.088** | - |

Significant values following Benjamini- Yekutieli correction for multiple tests in bold (p < 0.05), calculated using 50,000 bootstrap replications

**Table S6 – Pairwise *F*_ST_ with ENA correction for null alleles between *Ircinia campana* sites, calculated from 10 loci dataset**

|  | **GR** | **LK** | **BK** | **KC** | **WK** | **BC** | **LP** | **TA** | **SC** | **PAN1** | **PAN2** | **PAN3** | **MAY** | **BEQ** | **STV** | **STL** | **MAR1** | **MAR2** | **GU** |
| --- | --- | --- | --- | --- | --- | --- | --- | --- | --- | --- | --- | --- | --- | --- | --- | --- | --- | --- | --- |
| **GR** | - |  |  |  |  |  |  |  |  |  |  |  |  |  |  |  |  |  |  |
| **LK** | 0.111 | - |  |  |  |  |  |  |  |  |  |  |  |  |  |  |  |  |  |
| **BK** | 0.053 | 0.023 | - |  |  |  |  |  |  |  |  |  |  |  |  |  |  |  |  |
| **KC** | 0.135 | 0.017 | 0.055 | - |  |  |  |  |  |  |  |  |  |  |  |  |  |  |  |
| **WK** | 0.214 | 0.049 | 0.112 | 0.030 | - |  |  |  |  |  |  |  |  |  |  |  |  |  |  |
| **BC** | 0.104 | 0.006 | 0.028 | 0.033 | 0.055 | - |  |  |  |  |  |  |  |  |  |  |  |  |  |
| **LP** | 0.119 | 0.020 | 0.045 | 0.039 | 0.072 | 0.016 | - |  |  |  |  |  |  |  |  |  |  |  |  |
| **TA** | 0.226 | 0.166 | 0.171 | 0.172 | 0.219 | 0.158 | 0.176 | - |  |  |  |  |  |  |  |  |  |  |  |
| **SC** | 0.222 | 0.167 | 0.167 | 0.182 | 0.215 | 0.166 | 0.173 | 0.154 | - |  |  |  |  |  |  |  |  |  |  |
| **PAN1** | 0.233 | 0.160 | 0.176 | 0.175 | 0.225 | 0.165 | 0.168 | 0.139 | 0.142 | - |  |  |  |  |  |  |  |  |  |
| **PAN2** | 0.165 | 0.107 | 0.118 | 0.135 | 0.186 | 0.113 | 0.127 | 0.150 | 0.138 | 0.058 | - |  |  |  |  |  |  |  |  |
| **PAN3** | 0.190 | 0.161 | 0.167 | 0.173 | 0.235 | 0.158 | 0.168 | 0.139 | 0.150 | 0.046 | 0.054 | - |  |  |  |  |  |  |  |
| **MAY** | 0.204 | 0.132 | 0.143 | 0.162 | 0.183 | 0.136 | 0.154 | 0.152 | 0.146 | 0.186 | 0.135 | 0.161 | - |  |  |  |  |  |  |
| **BEQ** | 0.198 | 0.096 | 0.119 | 0.122 | 0.140 | 0.103 | 0.118 | 0.121 | 0.153 | 0.163 | 0.125 | 0.143 | 0.024 | - |  |  |  |  |  |
| **STV** | 0.218 | 0.128 | 0.143 | 0.138 | 0.162 | 0.127 | 0.139 | 0.122 | 0.172 | 0.163 | 0.141 | 0.139 | 0.048 | 0.016 | - |  |  |  |  |
| **STL** | 0.157 | 0.090 | 0.109 | 0.110 | 0.160 | 0.107 | 0.110 | 0.122 | 0.126 | 0.121 | 0.091 | 0.120 | 0.060 | 0.067 | 0.079 | - |  |  |  |
| **MAR1** | 0.127 | 0.065 | 0.086 | 0.095 | 0.152 | 0.071 | 0.085 | 0.113 | 0.128 | 0.090 | 0.074 | 0.112 | 0.119 | 0.094 | 0.113 | 0.051 | - |  |  |
| **MAR2** | 0.151 | 0.068 | 0.089 | 0.079 | 0.135 | 0.075 | 0.090 | 0.145 | 0.169 | 0.128 | 0.099 | 0.116 | 0.120 | 0.089 | 0.102 | 0.075 | 0.060 | - |  |
| **GU** | 0.167 | 0.110 | 0.124 | 0.122 | 0.180 | 0.099 | 0.124 | 0.140 | 0.149 | 0.160 | 0.119 | 0.151 | 0.153 | 0.123 | 0.151 | 0.107 | 0.057 | 0.098 | - |


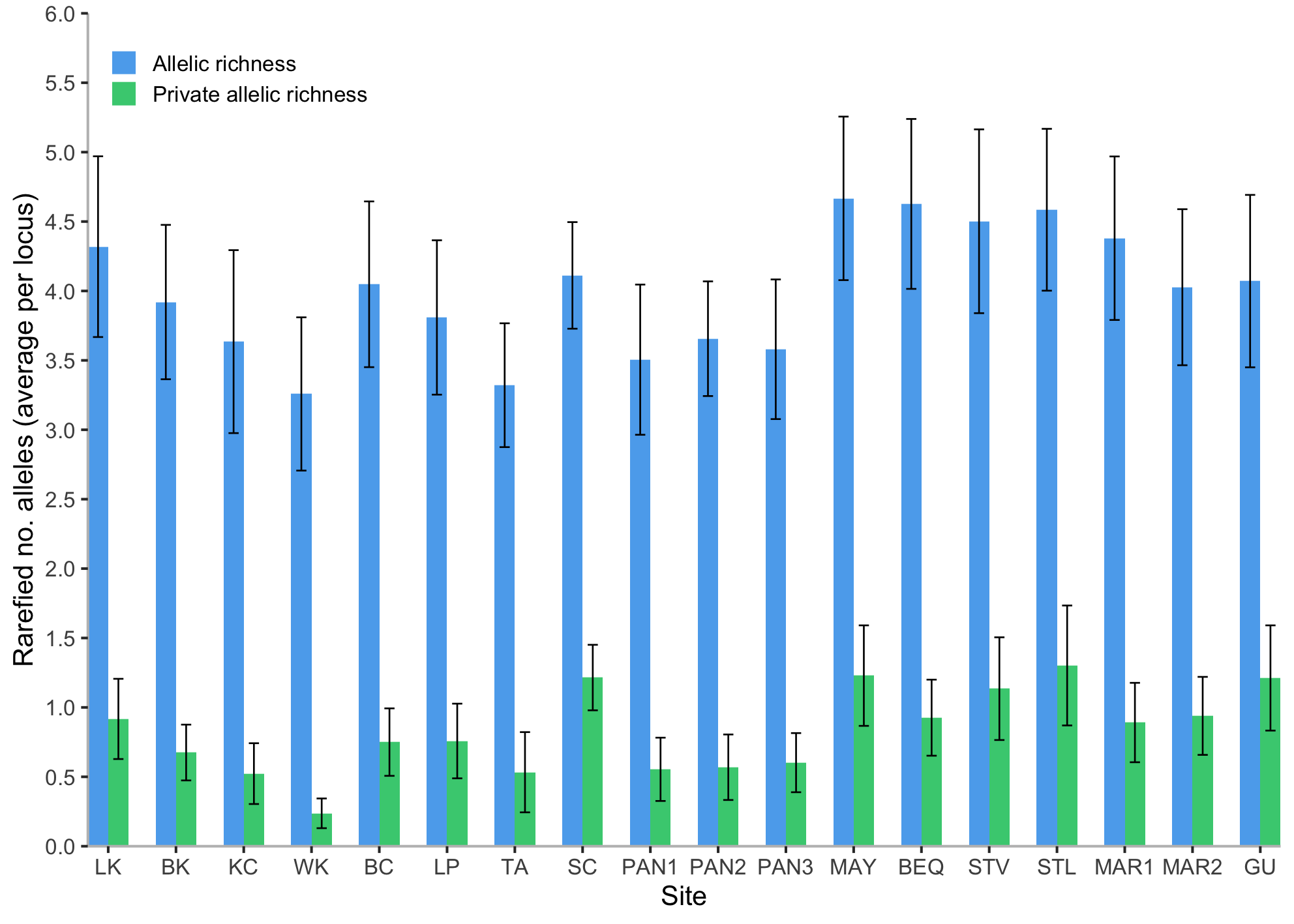


Fig. S1: Rarefied allelic richness and rarefied private allelic richness (maximum standardised sample size [max g] = 8) in *Ircinia campana* sites. Error bars +/- 1 SE.
